# Levels of evidence: a comparison between top medical journals and general pediatric journals

**DOI:** 10.1186/s12887-015-0324-9

**Published:** 2015-02-12

**Authors:** Dustin A Jacobson, Kunal Bhanot, Blake Yarascavitch, Jennifer Chuback, Ehud Rosenbloom, Mohit Bhandari

**Affiliations:** Michael G. DeGroote School of Medicine, McMaster University, Hamilton, ON Canada; Department of Neurosurgery, McMaster University, Hamilton, ON Canada; Department of Plastic Surgery, McMaster University, Hamilton, ON Canada; Depatment of Pediatrics, McMaster University, Hamilton, ON Canada; Departments of Epidemiology and Biostatistics and Orthopedic Surgery, McMaster University, Hamilton, ON Canada

**Keywords:** Evidence-based medicine, Data quality, Journal impact factor

## Abstract

**Background:**

Given the large number of publications in all fields of practice, it is essential that clinicians focus on the resources that provide the highest level of evidence (LOE). We sought to determine the LOE that exists in the field of pediatrics, present in the general pediatric as well as high impact clinical literature.

**Methods:**

Clinical pediatric literature, published between April 2011 and March 2012 inclusive in high-impact clinical journals (HICJ) (*New England Journal of Medicine, Journal of the American Medical Association, & The Lancet*) and the highest-impact general pediatric journals (GPJ) (*Pediatrics*, *Journal of Pediatrics*, & *Archives of Pediatrics & Adolescent Medicine*), was assessed. In addition to the LOE, articles were evaluated on criteria including subspecialty within pediatrics, number of authors, number of centers, and other parameters. Eligible level I randomized control trials were appraised using the Consolidated Standards of Reporting Trials (CONSORT) guidelines.

**Results:**

Of 6511 articles screened, 804 met inclusion criteria (68 in HICJ and 736 in GPJ). On average, LOE in pediatrics-focused articles within *The Lancet* were significantly higher than all GPJ (p < 0.05). Average CONSORT scores were significantly higher in HICJ vs. GPJ (15.2 vs. 13.6, respectively, p < 0.001).

**Conclusions:**

LOE and quality of randomized control trials within the pediatric field is highest within HICJ, however, only represent a small proportion of data published. Following CONSORT criteria, and promoting studies of high LOE may allow authors and readers to turn to journals and articles of greater clinical impact.

## Background

Evidence-based medicine purports to critically assess and utilize high-quality studies to help guide clinical practice. As the use of evidence-based medicine becomes increasingly popular both in and outside the academic sphere, supporting clinical practice with evidence-based decisions in the field of pediatrics is an important and evolving tenet of practice. At present, however, the quantity of literature published in pediatric medicine is voluminous, as it is in other medical fields. Clinicians do not often have the time to critically appraise and assess all relevant publications, and miss out on high-quality, relevant studies due to readership. Aside from selecting articles from ‘reputable journals’, no easily accessible and comprehensive screen is available for clinicians to help them decide which publications present studies of sound methodology and high level of evidence (LOE). Three other studies have done similar work in the fields of pediatric orthopedic surgery, neurosurgery and plastic surgery [1-3]. Namely, these works have suggested that higher levels of evidence and greater degrees of transparency in reporting for randomized control trials (RCTs) exist in higher impact clinical journals.

The objective of the current study is to determine the LOE that currently exists in the field of pediatrics. Specifically, this study compares the LOE of pediatric-related clinical studies found in general pediatric journals (GPJ) to that found in high impact clinical journals (HICJ) using the Oxford LOE guidelines, whereby the highest LOE includes RCTs and the lowest LOE includes studies that detail expert opinion/case series. Moreover, we compare randomized control trial design between these two groups.

We hypothesized that HICJ would have higher (closer to level I) mean LOE and higher CONSORT ratings (more fulfilled criteria), compared to GPJ. CONSORT criteria relate to study design and reporting of more criteria relate to better and higher quality RCTs [4-6].

## Methods

### Data sources and search methods/strategy

Journals with the highest impact factors in the English language, as reported by Thomas Reuters *Journal Citation Reports*, were chosen [7]. This study was designed to focus on clinically-oriented research; as such, journals emphasizing or exclusively publishing research from the basic sciences were excluded from this paradigm. General pediatric journals selected for review included *Pediatrics*, *Journal of Pediatrics* (*JofP*), and *Archives of Pediatrics & Adolescent Medicine* (*APAM*), as these journals represent the highest impact GPJ. In addition, general medical journals with total cite counts under 100,000 in the previous year were excluded to help ensure that journals reviewed would be of widespread appeal. Namely, journals excluded because of basic science output include *Nature* and *Science* and subspecialty journals were excluded to ensure generalizability. After this exclusion process, the three journals with the highest impact factors and highest cite counts were selected for review: *New England Journal of Medicine* (*NEJM*), *The Lancet* and *Journal of the American Medical Association* (*JAMA*).

### Inclusion and exclusion criteria

Studies screened included clinical papers that were human studies with a case series, case-control, cohort or RCT/metanalysis design. These studies had a focus in pediatrics and were published in English. We excluded non-English articles, basic science or cadaver studies, case reports, review articles of non-clinical cases and expert opinions without clinical examples. Study abstracts were assessed for initial eligibility (i.e. included if pediatric-focused). Once extracted, two reviewers assessed eligibility independently by hand searches of journal articles (DJ & KB); they were blinded to the other’s allocation. Discrepancies were discussed between reviewers to achieve consensus. Interobserver reliability was very good (kappa scores (κ) > 0.8).

### Data extraction and synthesis

Articles published between April 2011 and March 2012 were screened and categorized on the basis of journal, date of publication, subspecialty within pediatrics, number of authors, geographic region of corresponding author, number of centers used in trial, number of subjects/trials (latter used in meta-analyses), minimum age of entry of subjects, average age of entry of subjects, study subtype and LOE. In addition, the quality of reporting of eligible level I RCTs were appraised using the Consolidated Standards of Reporting Trials (CONSORT) guidelines [8]. Each eligible and included article was evaluated by two reviewers (DJ and KB). LOE allocation was completed using the Centre for Evidence Based Medicine’s Oxford Level of Evidence Guidelines, grouping subclasses within each LOE category (i.e. Ia, Ib, Ic become level I) [9]. In brief, higher level of evidence is closer to level I evidence (i.e. RCT is level I) and lower level of evidence is closer to level V evidence (i.e. expert opinion is level V). Reviewers were blinded to LOE allocation as well as CONSORT grading. Once the analysis was completed, any disagreements that occurred between reviewers were discussed until a consensus was reached.

Oxford LOE and CONSORT guidelines were used to examine top GPJ, as well as HICJ. The Oxford LOE guidelines are widely known and have been used effectively with individuals not trained in epidemiology with excellent interobserver reliability [3]. Likewise, the CONSORT guidelines represent the most widely used and accepted system for assessing RCTs, for their transparency of reporting [10].

### Statistical analysis

Data was collected using spreadsheets and imported for analyzed using SPSS 20.0 statistical software (IBM Corp, Armonk, NY). We used descriptive statistics to compare LOE between journals during the entire time period from April 2011 to March 2012. We compared the levels of evidence independently and grouped into high (Levels I and II) and low (Levels III and IV) using chi-square and students t-tests. Mean differences in LOE between journals was analyzed using one-way ANOVAs with Bonferroni post-hoc comparisons. We conducted a logistic regression analysis to determine the factors associated with higher or lower levels of evidence in the literature. All tests were one-tailed with a p value of <0.05 used as the conventional level of significance. To be sufficiently powered (i.e. beta = 0.20, alpha = 0.05, 80% study power) to identify a 0.5 absolute difference in mean LOE between high- and low-impact journals we required a total of 68 studies in each group. This is, indeed, an arbitrary cut-off. Because of the abstract nature of level of evidence being a numerical categorical variable, the conversion to an integer value for the purpose of comparisons of means has been done to aid in analysis and interpretation. There is support in the published literature for this type of interpretation and a mean difference of 0.5 being a useful cut-off [1,2].

### Ethics

No ethics approval was sought for this study or required by our research ethics board.

## Results

### Literature search

**Figure 1:**
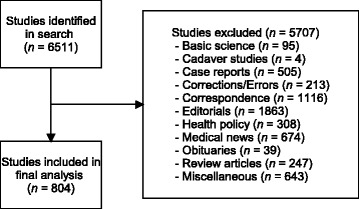
**Study flow diagram.**

Six thousand five hundred and eleven articles were screened, of which eight hundred and four papers met inclusion criteria (Figure 1). Twenty four articles (3.0%) were found in *JAMA*, twenty seven (3.4%) were found in *The Lancet*, seventeen (2.1%) were found in *NEJM*, one hundred and three (12.8%) were found in *APAM*, two hundred and fifty six (31.8%) were found in *JofP*, and three hundred and seventy seven (46.9%) were found in *Pediatrics* (Table 1).

**Table 1:** **Study characteristics**

	**Number of studies (%)**		**Number of studies (%)**
*Journal*		*Number of Authors*	
*JAMA*	24 (3.0)	<3	17 (2.1)
*Lancet*	27 (3.4)	3	89 (11.1)
*NEJM*	17 (2.1)	4	116 (14.4)
*APAM*	103 (12.8)	5	108 (13.4)
*J. Peds*	256 (31.8)	6	125 (15.5)
*Peds*	377 (46.9)	7	100 (12.4)
		>7	249 (31.0)
*Study type*			
Therapeutic	331 (41.2)	Number of Centers**	
Prognostic	403 (50.1)	1	605 (75.4)
Diagnostic	58 (7.2)	2	37 (4.6)
Economic & Decision Analysis	12 (1.5)	3	23 (2.9)
		4	15 (1.9)
Number of subjects*		5	10 (1.2)
<10	31 (3.9)	>5	112 (14.0)
11-50	109 (13.6)		
51-100	91 (11.3)	*Sub-Specialty*	
101-500	237 (29.6)	Adolescent Medicine	36 (4.5)
501-1,000	60 (7.5)	Pediatric Cardiology	47 (5.8)
1,001-5,000	131 (16.3)	Allergy & Immunology	39 (4.9)
5,001-10,000	38 (4.7)	Clinical Pharmacology	9 (1.1)
10,001-50,000	62 (7.7)	Pediatric Critical Care	17 (2.1)
50,001-100,000	9 (1.1)	Developmental Pediatrics	69 (8.6)
>100,000	34 (4.2)	Emergency Medicine	19 (2.4)
		Endocrinology & Metabolism	50 (6.2)
*Region*		Gastroenterology	28 (3.5)
North America	529 (65.8)	Hematology/Oncology	13 (1.6)
South America	7 (0.9)	Infectious Disease	67 (8.3)
Europe	174 (21.6)	Neonatal/Perinatal Medicine	68 (8.5)
Asia	29 (3.6)	Nephrology	13 (1.6)
Middle East	18 (2.2)	Neurology	35 (4.4)
India	2 (0.2)	Respirology	57 (7.1)
Africa	6 (0.7)	Rheumatology	8 (1.0)
Australia	39 (4.9)	Surgery	30 (3.7)
		General Pediatrics	141 (17.5)
		Other	58 (7.2)

*Number of subjects not reported in 2 studies, **Number of centers not reported in 2 studies.

JAMA – Journal of the American Medical Association, NEJM – New England Journal of Medicine, APAM – Archives of Pediatrics and Adolescent Medicine, J. Peds – Journal of Pediatrics, Peds – Pediatrics.

### Study characteristics

The majority of studies were pertinent to general pediatrics and the medical subspecialties (86.6%) with only a small minority related to pediatric surgery (all types) (3.7%) or emergency medicine (2.4%) (Table 1). Most articles originated from North America (65.8%) or Europe (21.6%). Studies from a single center (75.4%) were most common amongst those assessed, followed by multi-center studies with greater than five centers involved (14.0%). Level I studies had significantly more authors than level II studies (p = 0.001) or level IV studies (p = 0.009), but not significantly greater than level III studies (p = 0.098). Otherwise, no significance (p > 0.05) was found with all other study parameters in relation to the LOE.

### Level of evidence

**Figure 2:**
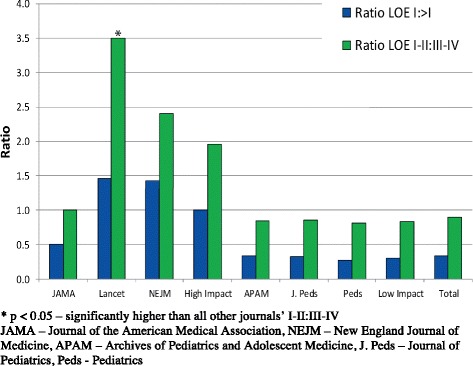
**Level of evidence (LOE) ratios by journal and category (high impact & low impact).**

Of 804 articles, 204 (25.4%) were graded level I, 175 (21.8%) were graded level II, 93 (11.6%) were graded level III, and 322 (41.3%) were graded level IV. Proportions of higher (LOE I and II) to lower (LOE III and IV) levels of evidence were calculated for each journal, as well as grouped proportions based on high impact vs. general pediatric literature. The highest ratio of high-to-low LOE was in *The Lancet* (3.5) followed by *NEJM* (2.4) with *JAMA* (1.0) and the GPJ journals having much lower ratios (Table 2 and Figure 2). Mean LOE by journal categories, as well as grouped based on HICJ and GPJ, show that LOE in *The Lancet* was significantly higher (i.e. closer to level I evidence) compared to all GPJ (*JoP* p = 0.001, *APAM* p = 0.002, *Pediatrics* p = 0.0001). *NEJM* had significantly higher LOE compared to *Pediatrics* (p = 0.027) and *JofP* (p = 0.039); however, it was not significantly higher than the average LOE found in *APAM* (p = 0.051). *JAMA* did not differ significantly in evidence compared to all other journals (*JoP* p = 0.255, *APAM* p = 0.261, *Pediatrics* p = 0.215, *Lancet* p = 0.657, *NEJM* p = 0.106).

**Table 2:** **Level of evidence by proportion and mean level of evidence based on journal**

***Journal***	***JAMA***		***Lancet***		***NEJM***		***APAM***		***J. Peds***		***Peds***		***Total***	
	**n**	**(%)**	**n**	**(%)**	**n**	**(%)**	**n**	**(%)**	**n**	**(%)**	**n**	**(%)**	**n**	**(%)**
*Level of evidence*														
I	8	(33.3)	16	(59.3^❖^)	10	(58.8^‡^)	26	(25.2^◆^)	63	(24.6^◆,*f*^)	81	(21.5^◆,*f*^)	204	(25.4)
II	4	(16.7)	5	(18.5)	2	(11.8)	21	(20.4)	55	(21.5)	88	(23.3)	175	(21.8)
III	5	(20.8)	3	(11.1)	3	(17.6)	9	(8.7)	25	(9.8)	48	(12.7)	93	(11.6)
IV	7	(29.2)	3	(11.1^◆^)	2	(11.8)	47	(45.6^❖^)	113	(44.1^❖^)	160	(42.4^❖^)	332	(41.3)
*Ratio LOE I-II:III-IV*	1.00		3.50		2.40		0.84		0.86		0.81		0.89	
*Mean LOE*	2.46		1.74^†^		1.82^∫^		2.75^*^		2.73^*,§^		2.76^*,§^		2.69	

^❖^Higher proportion compared to ^◆^(p < 0.05), ‡ Higher proportion compared to ^ƒ^(p < 0.05).

^†^Higher LOE compared to *(p < 0.01), ∫ Higher LOE compared to ^§^(p < 0.05).

JAMA – Journal of the American Medical Association, NEJM – New England Journal of Medicine, APAM – Archives of Pediatrics and Adolescent Medicine, J. Peds – Journal of Pediatrics, Peds – Pediatrics, LOE – Level of Evidence.

*The Lancet* and *NEJM* had higher proportions of level I & II evidence compared to all other journals (*Lancet vs JAMA* p = 0.02, *APAM* p = 0.0005, *JoP* p = 0.001, *Pediatrics* p = 0.003; *NEJM vs JAMA* p = 0.004, *APAM* p = 0.0007, *JoP* p = 0.0001, *Pediatrics* p = 0.0004) but did not differ significantly between each other (p = 0.47). As well, *The Lancet* was found to have significantly higher proportions of level I evidence compared to all GPJ (p = 0.03), and significantly lower proportions of level IV evidence compared to all GPJ (p = 0.02) (Table 2). This same result was not found with any other HICJ. When comparing grouped HICJ to GPJ, the proportion of level I evidence was significantly higher in HICJ (p = 0.002) (Table 3). Additionally, level IV evidence was lower in HICJ compared to GPJ (p = 0.03). Reliability in assessing kappa levels for LOE between the two reviewers measured 0.88.

**Table 3:** **Level of evidence and mean level of evidence based on journal type (i.e. high impact factor clinical journals vs. low impact factor general pediatric journals)**

***Journal***	***General clinic journals***		***Pediatric-specific journals***		***Total***	
	**n**	**(%)**	**n**	**(%)**	**n**	**(%)**
*Level of evidence*						
I	34	(50.0)^*^	170	(23.1)^*^	204	(25.4)
II	11	(16.2)	164	(22.3)	175	(21.8)
III	11	(16.2)	82	(11.1)	93	(11.6)
IV	12	(17.6)^*^	320	(43.5)^*^	332	(41.3)
*Ratio LOE I-II:III-IV*	1.96		0.83		0.89	
*Mean LOE*	2.01^†^		2.75^†^		2.69	

*p < 0.05, ^†^p < 0.01.

LOE – Level of Evidence.

Mean LOE – closer to 1 indicates RCT level and closer to 5 indicates expert opinion level.

### CONSORT grading

**Figure 3:**
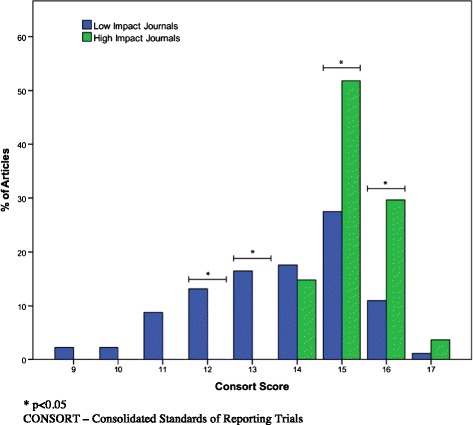
**CONSORT grading of articles based on journal type and total CONSORT score (out of 17).**

Although no significant differences in quality of reporting were found across journals in CONSORT checklist, average CONSORT scores were significantly higher in the grouped HICJ compared to GPJ (15.2 vs. 13.6, respectively, p = 0.001). Proportions of articles based on journal type (i.e. HICJ and GPJ) and CONSORT score can be found in Figure 3. Reliability in assessing kappa levels for CONSORT grading between the two reviewers measured 0.89.

## Discussion

This study demonstrates that studies with a higher LOE are more likely to be found in HICJ as opposed to GPJ. It is unclear why the HICJ differed in their LOE. One retrospective hypothesis was that researchers would preferentially submit higher LOE articles to journals with higher impact factors (i.e. *NEJM* and *The Lancet*), however, *The Lancet* seemed to have a trend towards highest LOE even compared to *NEJM.* It is still unclear why this difference is seen. It is possible that the difference relates to editorial differences between these and other journals. What is clear is that lower LOE exists in GPJ. The researchers in this study believe this is due to this aforementioned submission bias whereby authors are incentivized to submit and publish in HICJ. However, with such few studies published in HICJ, it is clear that the majority of information in terms of shear numbers used for evidence-based practice comes from GPJ. Therefore, it is crucial to encourage the submission and publication of good quality LOE and RCTs to GPJ. Moreover, while the minority of pediatric articles are published in HICJ, they are of good quality and should be read, referenced, and incorporated into practice.

RCT allocation used a subset of CONSORT guideline statements, chosen by the CONSORT Group to represent the minimum characteristics needed in reporting RCTs [8]. While reviewers did look at all aspects of the full article, the decision was made to use a subset of CONSORT criteria that examined what the CONSORT group and these researcher’s thought were the most important measures. Namely, these are measures that are easily identified by the reading public, and more importantly, a subset of minimum guidelines that the CONSORT group has identified for use in quickly and efficiently assessing transparency in reporting. It is well established that RCTs are at risk of bias and with this well-established and validated tool, although lower attributable bias cannot be guaranteed with its use, foreseeable risks are addressed [11]. Journals that implement these guidelines in their manuscript assessment process report higher quality RCTs [4-6].

When one looks only at the RCTs found in this analysis with the CONSORT criteria applied a similar trend to LOE is found. When grouped into GPJ and HICJ, HICJ have significantly higher fulfilled CONSORT criteria as compared to GPJ. These findings are consistent with findings by DeMauro *et al*. that in neonatal/infant subset populations of the pediatric population, quality of RCTs were higher in HICJ as compared to GPJ [12].

Looking at secondary characteristics, a trend of higher LOE based on increasing number of authors was noted. No significance was observed between the publication groups for subspecialty within pediatrics, geographic region of corresponding author, number of centers, number of subjects/trials, minimum age of entry of subjects, and average age of entry of subjects. Interestingly, the number of centers/subjects was not a predictor of high LOE. Additionally, age of entry of subjects and/or average age of subjects in trials did not correlate with LOE/CONSORT grading. This suggests that age is not a hindrance in designing and approving sound studies. As such, with a clearly demonstrated need for high-quality research in these ages [13,14], this finding negates excuses for poor methodology. This is not to say that research in children is not without limitations; with less prevalence of childhood disease [15] and smaller amounts of therapeutic agents designed for use in children [16], enrollment remains a challenge.

Our analyses suggest that better evidence is found in HICJ, but little is known regarding readership by pediatricians and other health care professionals. Therefore, although low in number, in order to not miss out on quality research, HICJ should be incorporated into the reference repertoire of practicing pediatricians and other health care professionals.

Our study has several limitations. First, it should be noted that our data does not allow for comment on any study-type excluded in our original search (i.e. basic science study) as LOE only applies to clinical studies. Additionally, neither our data nor our Oxford measuring tool discriminated between subdivisions within LOE (i.e. level Ia vs. Ib). This distinction within grades was not made in order to limit analysis to what would be considered realistic differences between LOE. However, our inter-observer agreement for both LOE and CONSORT were excellent (kappa scores (κ) > 0.8). This study should not imply that poor quality research is published in GPJ, rather, the comparison is important. Finally, our search and analysis was limited to a small subset of HICJ and GPJ. Therefore, further research may be needed to compare these two groups of literature outside of the clinical LOE.

## Conclusions

HICJ like *The Lancet* publish high LOE and well designed RCTs, however publish only a fraction of the amount of literature published by GPJ. Therefore, although important to incorporate HICJ into clinical repertoire, the vast majority of work published in GPJ should not be overlooked. Instead, raising LOE and RCT quality of all published work is the goal. Future research may look more at submission biases in favor of high impact medical journals.

Since the introduction of evidence-based medicine, efforts have been made to publish the highest quality research in publications considered to be at the pinnacle of their fields. By analyzing the most common journals in the field, this work more effectively addresses one of the basic pillars in medicine; being a lifelong learner and adapting one’s practice of medicine based on evolving knowledge and evidence.

## Abbreviations

RCT: Randomized control trial
LOE: Level of evidence
CONSORT: Consolidated Standards of Reporting Trials
GPJ: General pediatric journals
HICJ: High impact clinical journals
